# Evaluation of fluorescence imaging with indocyanine green in hepatocellular carcinoma

**DOI:** 10.1186/s40644-016-0064-6

**Published:** 2016-04-06

**Authors:** Masaki Kaibori, Kosuke Matsui, Morihiko Ishizaki, Hiroya Iida, Tatsuma Sakaguchi, Takumi Tsuda, Tadayoshi Okumura, Kentaro Inoue, Shingo Shimada, Seiji Ohtsubo, Mitsuo Kusano, Yuzuru Ikehara, Eiichi Ozeki, Tomoki Kitawaki, Masanori Kon

**Affiliations:** Department of Surgery, Hirakata Hospital, Kansai Medical University, 2-5-1 Shinmachi, Hirakata, Osaka 573-1010 Japan; Department of Surgery, Japan Labour Health and Welfare Organization Kushiro Rosai Hospital, Kushiro, Japan; Department of Oral and Maxillofacial Surgery, Japan Labor Health and Welfare Organization Kushiro Rosai Hospital, Kushiro, Japan; Department of Surgery, Seiwa Memorial Hospital, Sapporo, Hokkaido Japan; Research Centre for Medical Glycoscience, National Institute of Advanced Industrial Science and Technology, Tsukuba, Ibaraki Japan; Technology Research Laboratory, Shimadzu Corporation, Kyoto, Japan; Department of Mathematics, Hirakata Hospital, Kansai Medical University, Hirakata, Osaka Japan

**Keywords:** Hepatic resection, Hepatocellular carcinoma, Indocyanine green fluorescence imaging, Fibrosis stage

## Abstract

**Background:**

We hypothesized that indocyanine green (ICG) fluorescence patterns using Clairvivo OPT in resected liver specimens could confirm hepatocellular carcinoma (HCC) better than earlier commercial imaging systems. This preclinical trial evaluated the effectiveness of fluorescence imaging as an intraoperative cancer navigation tool.

**Methods:**

ICG fluorescence images of resected specimens from 190 patients with HCC were classified into two groups according to whether high fluorescence was seen in the HCC (high cancerous [HC] group) or in the surrounding liver tissue (high surrounding [HS] group). The HC and HS groups were sub-classified into whole and partial types and whole and ring types, respectively.

**Results:**

The HC group had significantly higher prevalence of esophageal or gastric varices, and worse liver function than patients in the HS group. The HC group also had a higher percentage of limited resection cases than did the HS group. Cirrhotic liver histology was significantly more common in the HC group than in the HS group. Multivariate analysis revealed that the HC group was a predictive factor for cirrhosis in HCC patients. Among the HC patients, a higher percentage of well-differentiated HCC cases were seen in the partial-type subgroup than in the whole-type subgroup (23/48 (48 %) vs. 7/68 (10 %)). In the HS group, the ring-type subgroup had a higher percentage of poorly differentiated HCC cases than did the whole-type subgroup (6/37 (16 %) vs. 0/37 (0 %)).

**Conclusion:**

Tumor differentiation and fibrosis in the non-cancerous liver parenchyma could affect ICG fluorescence imaging in HCC. ICG fluorescence imaging may be a good indication for fibrosis stage. In future, we will try to evaluate fluorescence imaging with ICG for intraoperative cancer navigation in HCC, using a portable near-infrared fluorescence imaging system.

## Background

Intraoperative fluorescent angiography is performed after intravenous (i.v.) injection of indocyanine green (ICG) to assess the patency of coronary artery bypass grafts [1–4]. Mitsuhashi et al. have reported that intraoperative fluorescent imaging during hepatobiliary surgery leads to better understanding of the anatomy of the hepatic arteries, portal vein, and bile ducts [5]. ICG binds to plasma proteins, and protein-bound ICG emits near-infrared light [6, 7]. In all the patients in our department, ICG is administered i.v. prior to surgery to measure the ICG retention rate at 15 min (ICGR15), to estimate the maximum limit of the hepatic volume to be resected safely [8, 9].

Human bile also contains proteins that bind to ICG [10], and we have recently reported that ICG fluorescent cholangiography can detect insufficiently closed bile duct stumps that cannot be identified by a standard bile leak test [11]. ICG fluorescence imaging of liver cancer has been used recently for intraoperative navigation [12]. We suggest that liver cancer can be identified by fluorescence imaging through visualization of the ICG that remains in the cancerous tissues and/or surrounding liver tissues after preoperative intravenous injection.

We hypothesized that ICG fluorescence patterns of resected HCC specimens using the “Clairvivo OPT,” which is a special-purpose imaging system for ICG, could detect hepatocellular carcinoma (HCC) better than earlier commercial imaging systems. The aim of this preclinical trial was to evaluate and develop the usefulness of fluorescence imaging as an intraoperative cancer navigation tool.

## Methods

### Patients

We retrospectively reviewed 190 patients with HCC who underwent R0 resection at our institution between January 2008 and December 2012. All patients provided written informed consent for participation in this study and the protocol was approved by the Institutional Ethics Review Board [The Institutional Review Board for Clinical Research of Kansai Medical University Hirakata Hospital (protocol identification number: H1303101). University hospital medical information network (UMIN) (protocol identification number: 000013112)].

### Clinicopathological variables and surgery

Before surgery, each patient underwent conventional liver function tests, and measurement of ICGR15. The fluorescent source was ICG (Diagnogreen; Daiichi Sankyo, Tokyo, Japan), which was injected at a dose of 0.5 mg/kg i.v. at 1–8 weeks prior to surgery. Hepatitis screening was done by measurement of hepatitis B surface antigen and hepatitis C antibody. The levels of α-fetoprotein and PIVKA-II (protein induced by vitamin K absence/antagonism-II) were also measured in all patients. Surgical procedures were classified according to the Brisbane terminology proposed by Strasberg et al. [13]. One senior pathologist reviewed each specimen for histological confirmation of the diagnosis.

### ICG fluorescence imaging of the resected specimens and fluorescent microscopy

The 242 HCC tumors were resected from a total of 190 patients, of whom 151 patients had solitary tumors, 30 patients had two tumors each, and 9 patients had three or more tumors. For patients with more than two tumors, we selected the tumor with the largest diameter to analyze by ICG fluorescence imaging.

In surgical specimens, near-infrared fluorescence images were taken by Clairvivo OPT (Shimadzu, Kyoto, Japan) using a filter set with excitation of 785 nm and emission of 845 nm. The system consisted of a horizontal sample table, excitation light sources, charge-coupled device (CCD) camera, camera lens, optical filter and white laser-emitting diode light sources illuminating the sample from four different angles. For excitation, five continuous-wave diode lasers emitting light at a wavelength of 785 nm and power of 0.07 mW/cm^2^ illuminated the whole object from five polar angles of 52, 120, 180, 240 and 308°. Each laser illuminated the object with a width of 40 mm and length of >120 mm. Fluorescence emission light was detected by a single CCD camera. An optical filter was placed in front of the CCD camera to cut the excitation light when the emission light was measured. The optical filter was a band-pass interference filter with a central wavelength of 845 nm and band width of 55 nm. The CCD sensor had 1024 × 1024 pixels, and 4 × 4 binning of the pixels provided 256 × 256 data for one CCD image [14].

**Fig. 1:**
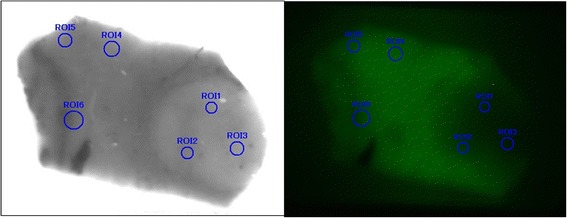
Quantification of fluorescence intensity in paraffin-embedded surgical specimens. Ratio is the average of fluorescence intensity at three points in the cancerous region (*ROI 1*, *2* and *3*)/average in three points in the non-cancerous surrounding liver tissue (*ROI 14*, *15* and *16*). *ROI* region of interest

Surgical specimens were fixed with formalin, sectioned at 10 mm, and stained with hematoxylin and eosin. Fluorescent microscopy was performed using an upright epifluorescence microscope (Eclipse 50i; Nikon Instruments, Tokyo, Japan) with a cooled CCD camera (Retiga EXi; QImaging, Surrey, BC, Canada), xenon light source (MAX-302; Asahi Spectra, Tokyo, Japan), and filters (excitation 775 ± 50 nm and emission 810 nm long pass and 845 ± 55 nm band pass; Chroma Technology, Rockingham, VT, USA). Fluorescent intensity in the paraffin-embedded surgical specimens was quantified in the cancerous and surrounding liver tissues (counts/mm^2^) (Fig. 1). The ratio was an average of fluorescent intensity at three points in the cancerous region/average in three points in the non-cancerous surrounding liver tissue. We analyzed the ratio initially in 110 patients; their median ratio was 2.5. For all 190 patients, those who showed high fluorescence in the HCC tissues (the high cancerous [HC] group, *n* = 116) and those with high fluorescence in their surrounding liver tissues (high surrounding [HS] group, *n* = 74), had ratios of fluorescent intensity in their cancerous/surrounding liver tissue of ≥2.5 and <2.5, respectively.

### Predictive factors

We performed univariate and multivariate analysis of 13 clinical factors to identify independent variables related to cirrhosis (stage F4) in liver histology of patients with HCC. The perioperative factors we investigated were age, sex, Child–Pugh class, alcohol abuse, etiology, presence or absence of esophageal and/or gastric varices, liver function [including ICGR15, albumin, total bilirubin, platelet count, aspartate aminotransferase (AST), and alanine aminotransferase (ALT)], and fluorescence imaging. All of the variables that were significant according to univariate analysis were examined with a Cox proportional hazards model to identify variables that had an independent influence on liver cirrhosis.

### Statistical analysis

Continuous variables were presented as the mean ± standard deviation (SD). Differences between two groups were assessed by the Mann–Whitney *U* test for results as the mean ± SD. Patients were divided into groups based on the median values of continuous variables (Table 4). Categorical data were compared with the *χ*^2^ test and Fisher’s exact test where appropriate. Factors that were found to be significant by univariate analysis were subjected to multivariate logistic regression analysis to determine their adjusted odds ratios (ORs). The OR was used to estimate relative risk of liver cirrhosis. The level of significance was set at *P* < 0.05. All statistical analyses were performed with SPSS for Windows version 11.0J (SPSS, Chicago, IL, USA).

## Results

Table 1 summarizes the preoperative characteristics of the two groups of patients with HCC. Patients in the HC group had a significantly higher prevalence of esophageal or gastric varices, and higher ICGR15, total bilirubin, AST and ALT, and lower platelet count than patients in the HS group.

**Table 1:** Preoperative clinical characteristics in the high cancerous (HC) and high surrounding (HS) groups

	HC group	HS group	*P*
	*n* = 116	*n* = 74	
Sex (male/female)	83/33	60/14	0.1377
Age (years)	68.8 ± 9.3	69.6 ± 9.3	0.5603
HBV/HCV/NBC	18/73/25	12/39/23	0.2953
Alcohol abuse (+)	25 (22 %)	14 (19 %)	0.6613
Esophageal and/or gastric varices (+)	23 (20 %)	1 (1 %)	**0.0002**
ICGR15 (%)	18.8 ± 10.5	12.8 ± 7.6	**<0.0001**
Days between injection of ICG and operation	25.5 ± 14.0	24.7 ± 12.3	0.7147
Platelet count (×10^4^/mL)	14.2 ± 6.2	19.6 ± 9.6	**<0.0001**
Total bilirubin (mg/dL)	0.83 ± 0.33	0.69 ± 0.23	**0.0016**
Albumin (g/dL)	3.7 ± 0.5	3.8 ± 0.5	0.5662
Prothrombin time (%)	89 ± 13	91 ± 11	0.2973
AST (U/L)	51 ± 27	37 ± 22	**0.0002**
ALT (U/L)	49 ± 34	38 ± 33	**0.0427**
AFP (ng/mL)	4103 ± 17,724	1829 ± 6357	0.3005
PIVKA-II (mAU/mL)	3476 ± 11,448	2761 ± 9618	0.6657

Values in parentheses are percentages. Data represent mean ± SD or number of patients

*AFP* α-fetoprotein, *HBV* hepatitis B virus, *HCV* hepatitis C virus, *NBC* non-hepatitis B or C virus

The data in bold was statistically significant

Table 2 shows that operating time, blood loss, and blood transfusion did not differ significantly between the two groups. The HC group had a higher percentage of limited resection cases than the HS group had. On pathological examination, tumor size, differentiation, microvascular invasion, number of tumors, and tumor stage did not differ significantly between the two groups. Cirrhotic liver histology was significantly more common in the HC group than in the HS group.

**Table 2:** Intraoperative and postoperative characteristics of the high cancerous (HC) and high surrounding (HS) groups

	HC group	HS group	*P*
	*n* = 116	*n* = 74	
Operating time (min)	349 ± 130	364 ± 136	0.423
Operative blood loss (mL)	1046 ± 1031	1021 ± 950	0.8915
Blood transfusion (+)	18 (16 %)	13 (18 %)	0.8247
Operative procedure			
Limited resection	69 (59 %)	22 (30 %)	**<0.0001**
Anatomic resection	47 (41 %)	52 (70 %)	
No. of patients with complications	16 (14 %)	8 (11 %)	0.5462
Tumor size (cm)	4.2 ± 3.5	5.2 ± 3.8	0.0798
Tumor differentiation			
Well	30 (26 %)	16 (22 %)	0.4813
Moderate	81 (70 %)	52 (70 %)	
Poor	5 (4 %)	6 (8 %)	
Microvascular invasion (+)	78 (67 %)	53 (72 %)	0.5246
Number of tumors			
Single	92 (79 %)	59 (80 %)	0.9444
Multiple	24 (21 %)	15 (20 %)	
Liver histology (fibrosis stage)			
F0	4 (3 %)	12 (16 %)	**<0.0001**
F1	8 (7 %)	36 (49 %)	
F2	10 (9 %)	25 (34 %)	
F3	36 (31 %)	0	
F4	58 (50 %)	1 (1 %)	
Tumor stage (TMN)			
I or II	58 (50 %)	28 (38 %)	0.1005
III or IV	58 (50 %)	46 (62 %)	

Values in parentheses are percentages. Data represent mean ± SD or number of patients

*F* fibrosis

The data in bold was statistically significant

### Factors affecting liver cirrhosis

Univariate analysis showed that factors associated with cirrhosis in patients with HCC were male sex (*P* = 0.0056), Child–Pugh class B (*P* = 0.0495), presence of esophageal and/or gastric varices (*P* < 0.0001), ICGR15 ≥14 % (*P* < 0.0001), albumin <3.9 g/dL (*P* = 0.0143), total bilirubin ≥0.7 mg/dl (*P* < 0.0001), platelet count <16 × 10^4^/μL (*P* < 0.0001), AST ≥39 IU/L (*P* = 0.0025), and high cancerous fluorescence imaging (*P* < 0.0001). Table 3 shows the results obtained by multivariate analysis of these factors. Esophageal and/or gastric varices, high cancerous fluorescence imaging, ICGR15 ≥14 %, and platelet count <1.6 × 10^4^/μL were identified as independent prognostic indicators of cirrhosis.

**Table 3:** Predictive factors for liver cirrhosis identified by multivariate analysis in patients with hepatocellular carcinoma

Variable	Odds ratio	95 % CI	*P*
Male sex	0.47	0.17–1.32	0.1523
Child–Pugh class	1.71	0.21–13.92	0.6161
Esophageal and/or gastric varices	4.02	1.11–14.58	**0.034**
High cancerous in fluorescence imaging	33.33	4.18–250	**0.0009**
ICGR15 ≥ 14 %	3.04	1.20–7.66	**0.0187**
Albumin <3.9 g/dL	1.11	0.44–2.77	0.8272
Total bilirubin ≥0.7 mg/dL	1.63	0.58–4.55	0.3512
Platelet <16 × 10^4^/mL	3.30	1.24–8.77	**0.0169**
AST ≥39 IU/L	1.33	0.52–3.38	0.5566

*CI* confidence interval

The data in bold was statistically significant

### Classification of positive fluorescence in cancerous or non-cancerous surrounding liver tissue regions

**Fig. 2:**
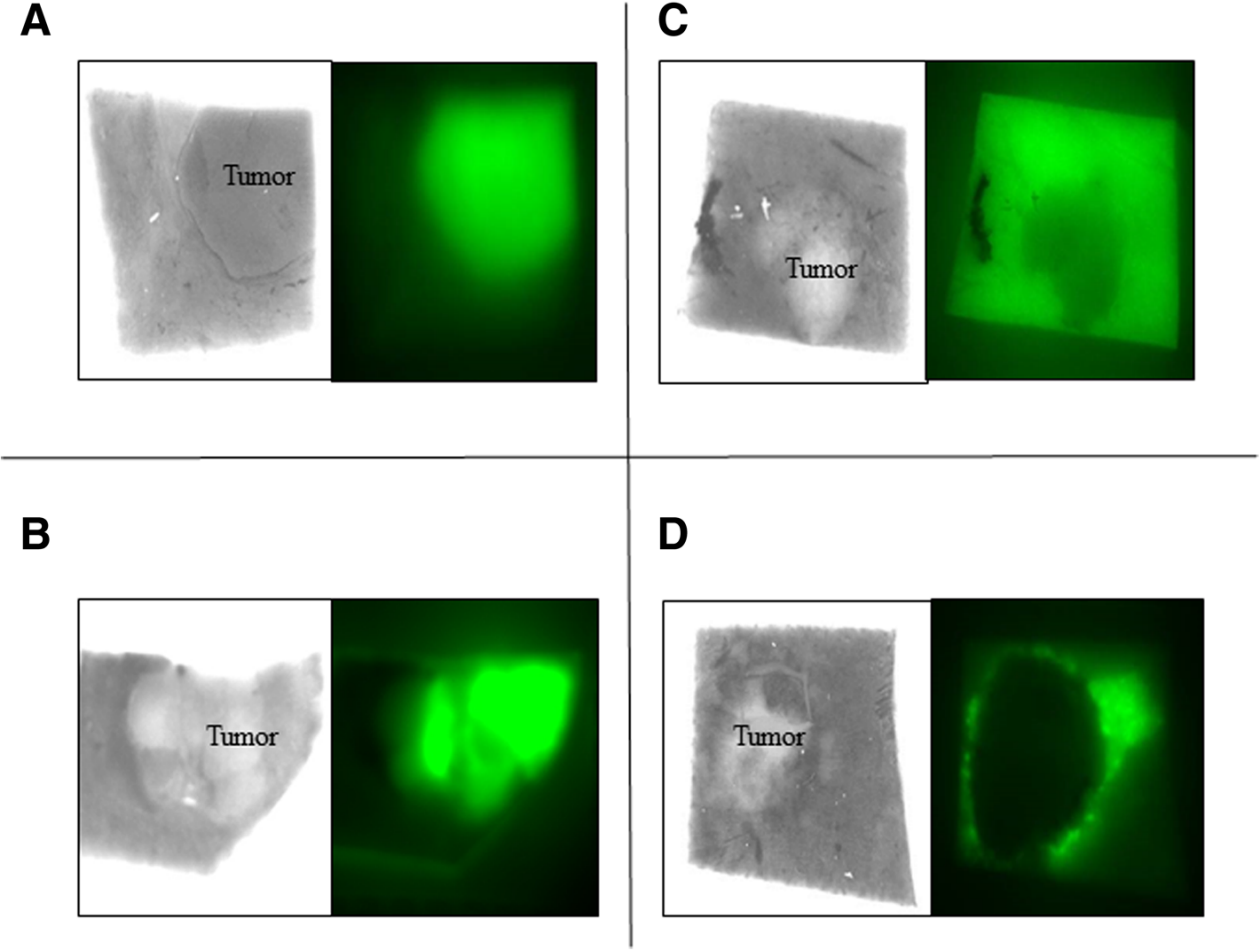
Whole (**a**) and partial (**b**) fluorescence patterns in the cancerous region. Whole- (**c**) and ring (**d**) fluorescence patterns in the non-cancerous surrounding liver tissue

We sub-classified the HC group into those with whole (Fig. 2a) or partial (Fig. 2b) types, and the HS group into those with whole (Fig. 2c) or ring types (Fig. 2d) types.

Preoperative characteristics, perioperative parameters and pathological findings did not differ significantly between the whole and partial types in the HC group. However, the partial type had a higher percentage of well-differentiated HCC cases than the whole type had. In addition, in the HS group, the ring type had a higher percentage of poorly differentiated HCC cases than the whole type had (Table 4).

**Table 4:** Pathological tumor characteristics of whole and partial fluorescence patterns in the high cancerous group, and whole and ring patterns in high surrounding group ICG fluorescence of HCC

High cancerous group	Whole type (*n* = 68)	Partial type (*n* = 48)	*P*
Tumor differentiation	7/59/2	**23/**22/3	**<0.0001**
(well/moderate/poor)	(10 %/87 %/3 %)	(**48 %**/46 %/6 %)	
High surrounding group	Whole type (*n* = 37)	Ring type (*n* = 37)	
Tumor differentiation	8/29/0	8/23/**6**	**0.0352**
(well/moderate/poor)	(22 %/78 %/0)	(22 %/62 %/**16 %**)	

Data represent number (percentage) of patients

The data in bold was statistically significant

### Fluorescent microscopy

**Fig. 3:**
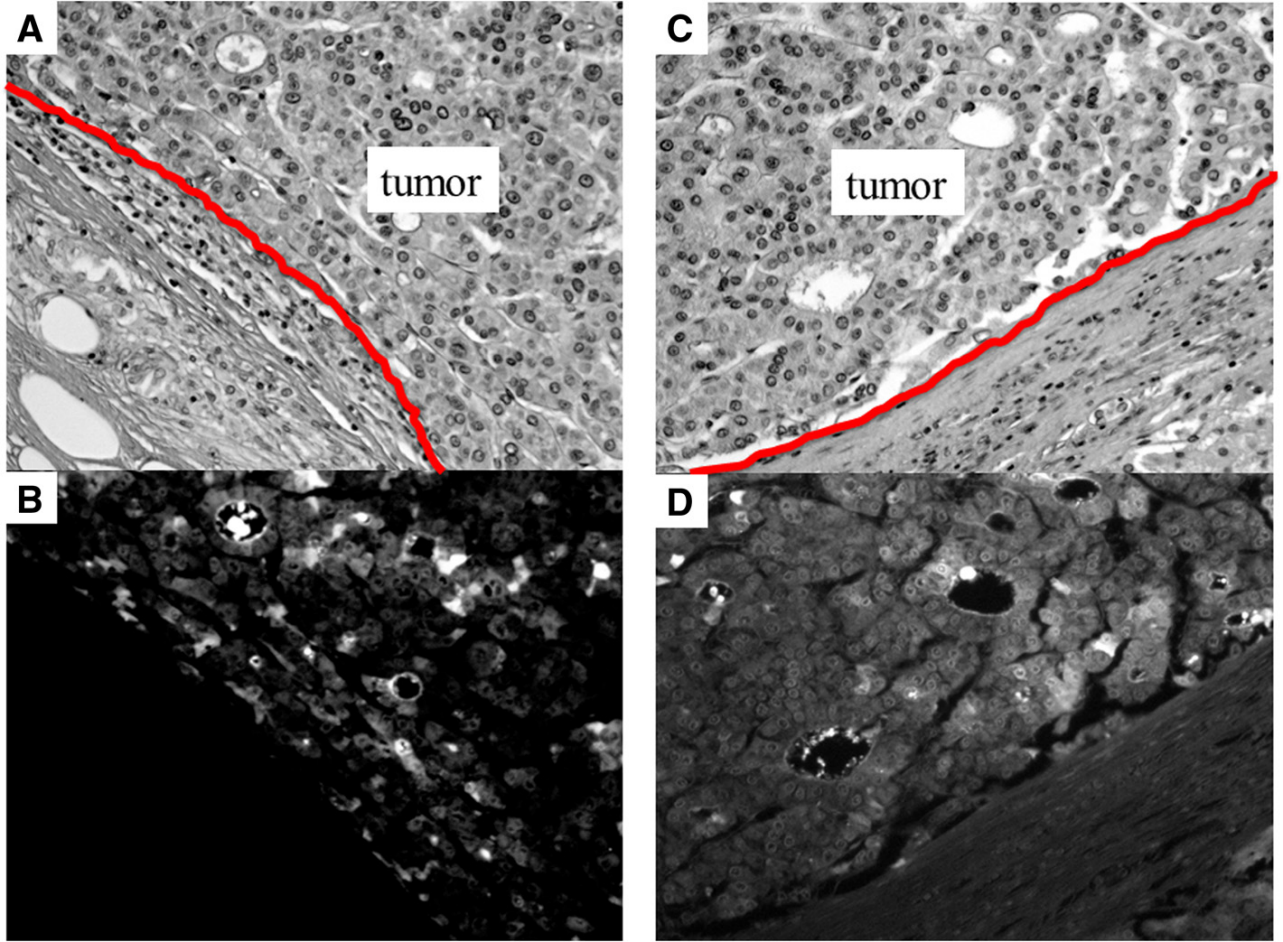
Fluorescent microscopy in two cases with positive fluorescence in the cancerous region. (**a** and **c**) Hematoxylin–eosin staining. (**b** and **d**) Fluorescent microscopy. ICG fluorescence was identified at the canalicular side of the cancer cell cytoplasm and pseudoglands of the HCC

**Fig. 4:**
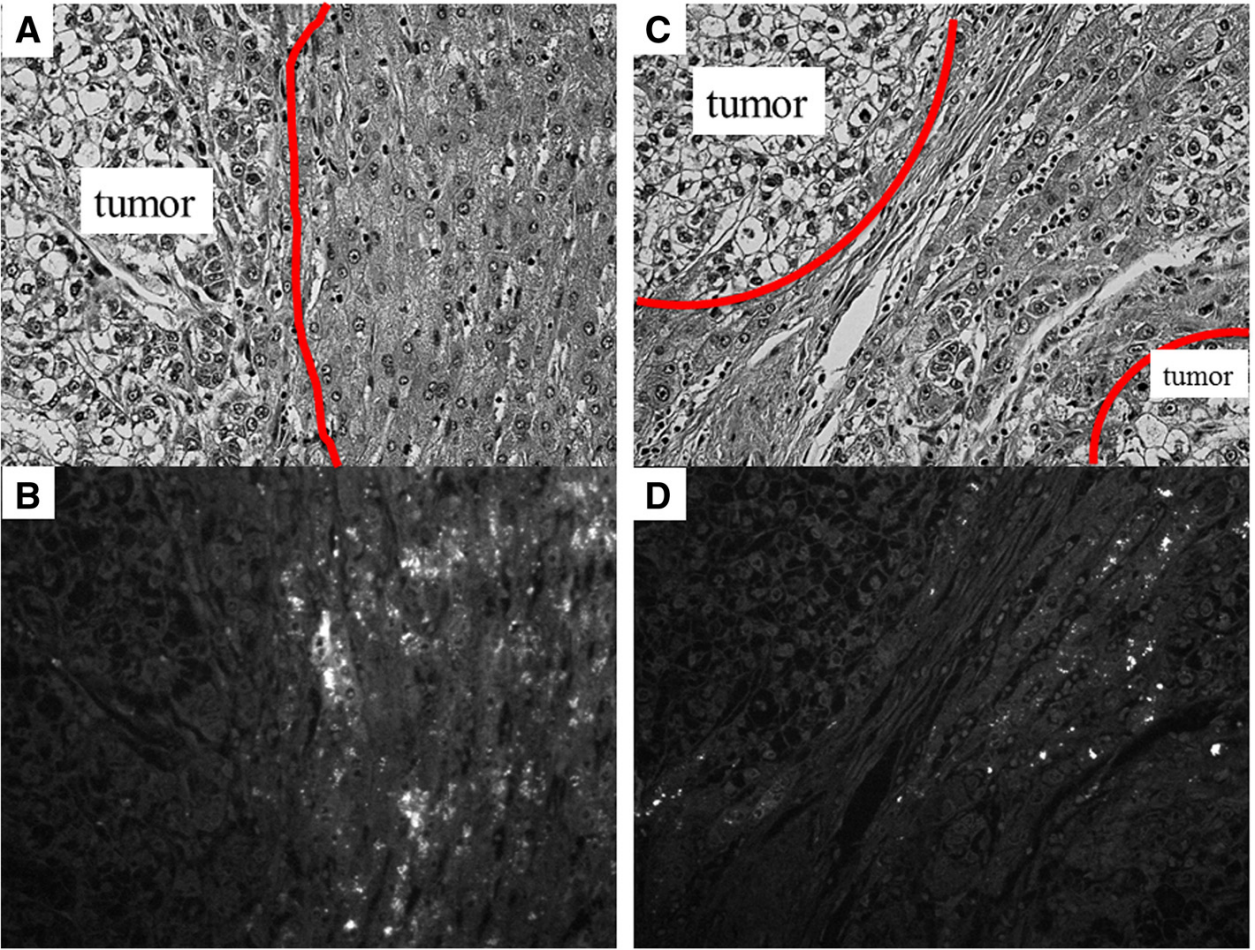
Fluorescent microscopy in two cases with positive fluorescence in the surrounding liver tissue region. (**a** and **c**) Hematoxylin–eosin staining. (**b** and **d**) Fluorescent microscopy. Most fluorescence was confirmed in the non-cancerous surrounding liver tissues

ICG fluorescent imaging confirmed that significant fluorescence was demonstrated in the cancerous or non-cancerous surrounding liver regions of the two surgical specimens (Figs. 3 and 4). In the HC group, ICG fluorescence was identified at the canalicular side of the cancer cell cytoplasm and pseudoglands of HCC (Fig. 3). In the HS group, most fluorescence was confirmed in the surrounding non-cancerous liver tissues (Fig. 4).

## Discussion

We classified surgical specimens into two groups according to ICG fluorescence patterns in the cancerous and non-cancerous surrounding liver parenchyma (HC and HS groups). The HC group had significantly more advanced fibrosis in the non-cancerous liver parenchyma than the HS group had. Multivariate analysis revealed that fluorescence imaging in the HC group was a prognostic factor for cirrhosis in HCC patients. In the HC group, the partial type had a higher percentage of well-differentiated HCC cases than the whole type had. Previous reports [15, 16] showed that preoperatively injected ICG did not pool sufficiently in the tumor, as the arterial supply in well-differentiated HCC was less than in moderately or poorly differentiated HCC.

Recently Ishizawa et al. [17] reported the mechanisms of ICG accumulation in liver cancer tissues. They demonstrated that preserved portal uptake of ICG in differentiated HCC cells by Na^+^/taurocholate co-transporting polypeptide 8 and organic anion-transporting polypeptide 8, with concomitant biliary excretion disorders, causes accumulation of ICG in cancerous tissues after preoperative i.v. administration. They classified resected specimens from 170 HCC patients into two patterns: ICG fluorescence in the cancerous tissues (cancerous-type fluorescence); and only in the surrounding non-cancerous liver parenchyma (rim-type fluorescence). The cancerous-type fluorescence in HCC was associated with higher cancer cell differentiation as compared with the rim-type HCC. In the background characteristics of their patients with HCC, cirrhotic liver histology and preoperative ICGR15 in the cancerous-type HCC were more common [101/240 (42 %) vs. 8/33 (24 %)] and higher [median (ranges): 13.0 (3.3–46.5) vs. 10.4 (3.3–29.6)], respectively, than those in the rim-type. However, the difference was not significant. The number of patients with Child–Pugh class B or C was greater in cancerous-type fluorescence HCC than in rim-type [11/240 (5 %) vs. 0/33 (0 %)]. We also found that the ring type in the high non-cancerous group had a higher percentage of poorly differentiated HCC cases than the whole type had.

The interval between injection of ICG and day of operation did not differ significantly between our two groups: the average interval was 25.5 and 24.7 days in the HC and HS groups, respectively. No studies have clearly identified the optimal ICG concentration or interval between ICG injection and surgery for liver tumors. Kaneko et al. [18] reported that ICG was preferentially taken up by human hepatoma tumors (HuH-7 and HepG2) transplanted subcutaneously into nude mice. The ICG remained in the HuH-7 tumor for a long time, maintaining a high tumor-to background ratio for at least 6 days after ICG administration. However, in clinical situations, the pharmacokinetics regarding retention and disappearance of ICG in HCC has not been resolved in detail. It is unclear whether there is any relation between fluorescence intensity of ICG in the tumor and fibrosis in the non-cancerous liver parenchyma. It is necessary to determine the optimal concentration of ICG and the interval between ICG injection and operation for suitable fluorescence imaging of the tumor.

## Conclusion

Several near-infrared fluorescence imaging devices that use various doses of ICG for clinical application have been developed [19]. An advanced device for simultaneous capturing of color and near-infrared images using a laser-emitting diode and color CCD camera is being developed as a surgery support system.

In conclusion, ICG fluorescence imaging for HCC is effective for measuring tumor differentiation as well as fibrosis detection in non-cancerous liver parenchyma. ICG fluorescence imaging may be a good indicator of the stage of fibrosis. Further detailed analysis by a higher-resolution apparatus may be useful for fluorescence imaging as an intraoperative cancer navigation tool. In the immediate future, we will try to evaluate the fluorescence imaging with ICG for intraoperative cancer navigation in HCC using the near-infrared fluorescence imaging system for simultaneous capturing of color and near-infrared images.
